# Unseen patterns of preventable emergency care: Emergency department visits for ambulatory care sensitive conditions

**DOI:** 10.1177/13558196211059128

**Published:** 2022-02-06

**Authors:** Beth Parkinson, Rachel Meacock, Katherine Checkland, Matt Sutton

**Affiliations:** 1 Health Organisation, Policy and Economics (HOPE) Research Group, Centre for Primary Care and Health Services Research, 5292 The University of Manchester, UK; 2 Melbourne Institute: Applied Economic and Social Research, 5292 University of Melbourne, Australia

**Keywords:** ambulatory care sensitive conditions, preventable emergency care, international classification of diseases coding

## Abstract

**Objective:**

Admissions for ambulatory care sensitive conditions (ACSCs) are often used to measure potentially preventable emergency care. Visits to emergency departments with ACSCs may also be preventable care but are excluded from such measures if patients are not admitted. We established the extent and composition of this preventable emergency care.

**Methods:**

We analysed 1,505,979 emergency department visits (5% of the national total) between 1 April 2015 and 31 March 2017 at six hospital Trusts in England, using International Classification of Diseases diagnostic coding. We calculated the number of visits for each ACSC and examined the proportions of these visits that did not result in admission by condition and patient characteristics.

**Results:**

11.1% of emergency department visits were for ACSCs. 55.0% of these visits did not result in hospital admission. Whilst the majority of ACSC visits were for acute rather than chronic conditions (59.4% versus 38.4%), acute visits were much more likely to conclude without admission (70.3% versus 33.4%). Younger, more deprived and ethnic minority patients were less likely to be admitted when they visited the emergency department with an ACSC.

**Conclusions:**

Over half of preventable emergency care is not captured by measures of admissions. The probability of admission at a preventable visit varies substantially between conditions and patient groups. Focussing only on admissions for ACSCs provides an incomplete and skewed picture of the types of conditions and patients receiving preventable care. Measures of preventable emergency care should include visits in addition to admissions.

## Introduction

Avoidable health care utilization is of growing concern as it represents an unnecessary use of resources and is often regarded as symptomatic of suboptimal community and primary health care.^1-9^ Emergency admissions for ambulatory care sensitive conditions (ACSCs) are frequently used as a measure of potentially preventable hospitalizations.^1^ These include chronic conditions such as asthma and diabetes, where good quality care should prevent exacerbations; acute conditions such as dehydration and gastroenteritis, where timely and effective care stops the condition deteriorating; and vaccine preventable conditions.^2^

Admissions for ACSCs have been validated as performance indicators in many countries, such as the US, Germany, Spain and the UK.^3-6^ The Organization for Economic Co-operation and Development reports rates of ‘avoidable admissions’ from all 34 member countries as indicators of the quality of primary care.^7^ Systematic reviews show that physician supply and longitudinal continuity of care reduce avoidable admissions for chronic conditions^8^ and that better access to primary health care reduces ACSC admission rates.^9^

However, focussing only on admissions will not capture all clinically preventable emergency care. This is because only a subset of patients visiting an emergency department (ED) are admitted, and their composition and patient characteristics may be different from non-admitted attendances. Consequently, policies to reduce preventable utilization may not be targeting all conditions or patients who experience preventable episodes of emergency care.

ACSC ED visits have so far only been studied in the US context,^10-12^ where variations in insurance coverage confound comparisons across population groups. Furthermore, US EDs act as ‘safety net’ providers for those without insurance or a regular doctor. We focus here on EDs in England which are available to all, free at the point of access.

The prevalence of potentially preventable ED visits in England has not been examined due to limitations in how ED data are recorded. ACSCs are identified using international classification of diseases (ICD) diagnosis codes, but most EDs in England use a diagnosis classification system that is too broad to code preventable visits in this way.

However, we identified six National Health Service (NHS) hospital Trusts in England that do in fact use ICD classification in their EDs. For the first time, this enables identification of ED attendances for ACSCs in England and therefore an estimate of potentially preventable attendances. We also used these data to establish the extent and composition of preventable emergency care missed when focussing only on admissions. We also examined the proportions of preventable visits not resulting in admission across conditions and patient characteristics.

## Methods

### Data

We used patient-level data on all visits to major (known as ‘type 1’) EDs in England from Hospital Episode Statistics between 1 April 2015 and 31 March 2017 inclusive.^13^ We obtained the following information from each visit record: patient age, gender, ethnicity, area of residence, arrival mode, diagnosis and whether the visit resulted in admission to hospital. We attached quintiles of the Index of Multiple Deprivation 2019^14^ to the patient’s lower layer super output area of residence.^15^

### Identification of potentially preventable ED visits

There is no universally agreed definition of ACSCs.^2^ We used the definition of ACSCs used in both the NHS Outcomes Framework and Clinical Commissioning Group (CCG) Outcomes Indicator Set, since these admissions are used to measure performance in the English NHS.^16-17^ We used the 19 conditions contained in two CCG Outcome Indicator Set indicators: ‘Indicator 2.6 Unplanned hospitalizations for chronic ambulatory care sensitive conditions’,^16^ and ‘Indicator 3.1 Emergency admissions for acute conditions that should not usually require hospital admission’ which includes acute and vaccine preventable conditions.^17^ The codes and full descriptions are listed in the Online Supplement 1 Table S1.

Hospitals can report up to 12 diagnoses fields on ED visit records, but 94.5% of visits have only one diagnosis. In the main analysis, we classified visits only using the first diagnosis field. In two sensitivity checks, we first analysed only visits with one diagnosis field completed and then we classified visits as preventable if any diagnosis field was an ACSC.

### Identification of hospital Trusts using ICD diagnoses in their EDs

Hospitals in England can record diagnoses based on three coding schemes: Accident and Emergency diagnosis, Read Coded Clinical Terms and ICD-10.^18^ The coding system is determined by the software in each hospital’s patient administration system. As ACSCs are identified using ICD codes, we restricted our sample to hospitals that only used this classification system and had few missing diagnoses.

We checked the coding of diagnoses using Stata’s ‘icd10 check’ command^19^ in data from all 140 hospital Trusts in England. We identified nine Trusts where over 99% of visits with non-missing diagnosis fields contained a valid ICD code. We then ranked these Trusts by levels of missing diagnosis data. We selected the six Trusts with less than 12.5% missing data, as this was a natural cut-off point since the extent to which data were missing was almost twice as high (24.7%) in the next Trust. Our chosen cut-off was approximately half the extent to which data was missing in diagnostic coding in the Trusts not using the ICD classification system (25.7%).

In total, the six included Trusts recorded 1,505,979 ED visits over the 2 years, representing 4.9% of total visits in England across the same period. Two of the six Trusts are in the West Midlands region of England, three are in the South West, and one is in the South East of England. Online Supplements 2 and 3 provide details of data cleaning, levels of missing diagnosis and results of Stata’s ‘icd10 check’ command.

### Statistical methods

First, we examined how the visits at the six selected Trusts compared to those at Trusts in the rest of England in terms of patient age, gender, area deprivation, arrival mode and discharge method.

We then estimated the prevalence of preventable ED visits at the six hospital Trusts as measured by the number of ACSC visits. We report this in absolute volumes and as a proportion of all ED visits. We classified the preventable visits into three groups: chronic, acute or vaccine preventable. We then examined which of the 19 preventable conditions were most prevalent. We also stratified this by age group.

Finally, we explored the characteristics of patients whose preventable care would be missed if only *admissions* had been counted. We examined how the proportion of ACSC visits that did not result in admission varied across patient age group, gender, ethnicity, area deprivation and presenting condition.

## Results

Visits at the six selected hospital Trusts had broadly similar characteristics as visits at all other Trusts in England (Online Supplement 4 Table S4). A higher proportion of visits at the six selected Trusts were by patients living in the most deprived areas (35.7% versus 27.4%).

As Table 1 shows, 11.1% of ED visits (*n*=158,266) at the six hospital Trusts were recorded as resulting primarily from an ACSC. Of these preventable visits, 38.4% were for chronic conditions, 59.4% were for acute episodes and 2.3% were for flu or other vaccine preventable conditions.

**Table 1.** Emergency department visits for ambulatory care sensitive conditions, by hospital Trust.

	All 6 trusts		Trust 1		Trust 2		Trust 3		Trust 4		Trust 5		Trust 6	
	N	%	N	%	N	%	N	%	N	%	N	%	N	%
Total attendances	1,430,203		141,805		145,960		258,190		206,870		489,800		187,575	
Non-ACSC^a^	1,271,937	88.9%	129,545	91.4%	130,275	89.3%	227,965	88.3%	188,915	91.3%	440,385	89.9%	154,850	82.6%
ACSC	158,266	11.1%	12,260	8.6%	15,690	10.7%	30,225	11.7%	17,960	8.7%	49,415	10.1%	32,720	17.4%
	N	% of ACSC visits	N	% of ACSC visits	N	% of ACSC visits	N	% of ACSC visits	N	% of ACSC visits	N	% of ACSC visits	N	% of ACSC visits
Chronic ACSC	60,709	38.4%	4820	39.3%	5585	35.6%	12,430	41.1%	7375	41.1%	21,410	43.3%	9090	27.8%
Acute ACSC	93,955	59.4%	6885	56.2%	10,050	64.1%	16,460	54.5%	9610	53.5%	27,415	55.5%	23,540	71.9%
Vaccine preventable conditions	3602	2.3%	555	4.5%	50	0.3%	1335	4.4%	975	5.4%	595	1.2%	95	0.3%
By condition	N	% of ACSC visits	N	% of ACSC visits	N	% of ACSC visits	N	% of ACSC visits	N	% of ACSC visits	N	% of ACSC visits	N	% of ACSC visits
Cellulitis	24,817	15.7%	1650	13.5%	2500	15.9%	3805	12.6%	3810	21.2%	10,680	21.6%	2370	7.2%
Ear, nose and throat conditions	24,665	15.6%	1000	8.2%	2225	14.2%	4095	13.5%	1165	6.5%	5045	10.2%	11,140	34.0%
Angina	16,980	10.7%	1075	8.8%	1095	7.0%	3420	11.3%	1395	7.8%	6845	13.9%	3145	9.6%
Dehydration and gastroenteritis	16,059	10.1%	1480	12.1%	1460	9.3%	1635	5.4%	1650	9.2%	5605	11.3%	4230	12.9%
Asthma	12,131	7.7%	840	6.9%	1045	6.7%	2330	7.7%	1435	8.0%	4670	9.5%	1810	5.5%
Urinary tract infections	11,420	7.2%	230	1.9%	2475	15.8%	4560	15.1%	430	2.4%	1020	2.1%	2705	8.3%
Convulsions	10,528	6.7%	1775	14.5%	730	4.7%	1215	4.0%	1820	10.1%	3755	7.6%	1235	3.8%
Chronic obstructive pulmonary disease	9329	5.9%	955	7.8%	775	4.9%	2200	7.3%	1320	7.3%	2915	5.9%	1165	3.6%
Atrial fibrillation	6144	3.9%	925	7.5%	760	4.8%	1305	4.3%	930	5.2%	1420	2.9%	800	2.4%
Epilepsy	6104	3.9%	185	1.5%	765	4.9%	1580	5.2%	1065	5.9%	1485	3.0%	1025	3.1%
Dental conditions	4843	3.1%	460	3.8%	425	2.7%	1080	3.6%	720	4.0%	1280	2.6%	875	2.7%
Congestive heart failure	4391	2.8%	550	4.5%	620	4.0%	1130	3.7%	520	2.9%	1085	2.2%	490	1.5%
Diabetes complications	4083	2.6%	100	0.8%	355	2.3%	465	1.5%	630	3.5%	2030	4.1%	500	1.5%
Flu and pneumonia	3525	2.2%	555	4.5%	45	0.3%	1335	4.4%	975	5.4%	590	1.2%	25	0.1%
Perforated ulcer	1623	1.0%	295	2.4%	235	1.5%	75	0.2%	10	0.1%	25	0.1%	985	3.0%
Hypertension	1262	0.8%	145	1.2%	110	0.7%	0	0.0%	0	0.0%	900	1.8%	110	0.3%
Dementia	250	0.2%	50	0.4%	30	0.2%	0	0.0%	75	0.4%	60	0.1%	35	0.1%
Other vaccine preventable conditions	77	0.0%	0	0.0%	*	*	0	0.0%	0	0.0%	*	*	65	0.2%
Anaemia	35	0.0%	0	0.0%	*	*	0	0.0%	0	0.0%	0	0.0%	*	*

Due to Hospital Episode Statistics data disclosure, the trust level data volumes were rounded to nearest 5. Volumes of 7 or under, and their associated percentages, were supressed, represented by *.

^a^ACSC: Ambulatory care sensitive condition.

Restricting to visits with no secondary diagnoses recorded resulted in 11.0% (149,109/1,352,135) of visits classified as preventable. 11.5% of visits were classified as preventable when we considered any diagnosis field (Online Supplement 5 Table S5).

Returning to Table 1, five of the 19 ACSCs accounted for almost 60% of preventable visits. These were cellulitis (24,817 visits, 15.7% of all ACSC visits); ear, nose and throat infections (24,665, 15.6%); angina (16,980, 10.7%); dehydration and gastroenteritis (16,059, 10.1%); and asthma (12,131, 7.7%). The five least frequent ACSCs were anaemia (35, 0.0%), other vaccine preventable conditions (77, 0.0%), dementia (250, 0.2%), hypertension (1,262, 0.8%), and perforated ulcer (1623, 1.0%).

As shown in Table 2, the age groups with the highest proportions of attendances for ACSCs were ages 0–4 (18.3%), ages 65–84 (14.8%), and ages 85+ (13.8%) compared to 7.9% in ages 16–44. Young children (0–4 years of age) were much more likely to attend for an acute condition (90.4% of ACSCs), whilst in older age groups chronic conditions made up a larger proportion of preventable visits (61.7% of ACSC attendance by individuals aged 65 to 84, and 54.2% of attendances by individuals aged 85+).

**Table 2.** Emergency department visits for ambulatory care sensitive conditions, by age group.

	Age 0 to 4		Age 5 to 15		Age 16 to 44		Age 45 to 64		Age 65 to 84		Age 85+	
	N	%	N	%	N	%	N	%	N	%	N	%
Total	144,325		165,622		525,798		272,803		236,788		84,867	
Non-ACSC^a^	117,849	81.7%	151,739	91.6%	484,205	92.1%	243,185	89.1%	201,786	85.2%	73,173	86.2%
ACSC	26,476	18.3%	13,883	8.4%	41,593	7.9%	29,618	10.9%	35,002	14.8%	11,694	13.8%
	N	% of ACSC visits	N	% of ACSC visits	N	% of ACSC visits	N	% of ACSC visits	N	% of ACSC visits	N	% of ACSC visits
Chronic	2405	9.1%	4053	29.2%	11,253	27.1%	15,063	50.9%	21,595	61.7%	6340	54.2%
Acute	23,935	90.4%	9761	70.3%	29,838	71.7%	13,869	46.8%	12,008	34.3%	4544	38.9%
Vaccine preventable conditions	136	0.5%	69	0.5%	502	1.2%	686	2.3%	1399	4.0%	810	6.9%
By condition	N	% of ACSC visits	N	% of ACSC visits	N	% of ACSC visits	N	% of ACSC visits	N	% of ACSC visits	N	% of ACSC visits
Cellulitis	1330	5.0%	1508	10.9%	9924	23.9%	6305	21.3%	4409	12.6%	1341	11.5%
Ear nose and throat conditions	12,701	48.0%	4038	29.1%	6103	14.7%	1203	4.1%	526	1.5%	94	0.8%
Angina	0	0.0%	3	0.0%	1673	4.0%	6196	20.9%	7169	20.5%	1939	16.6%
Dehydration and gastroenteritis	4952	18.7%	1950	14.0%	3860	9.3%	1790	6.0%	2433	7.0%	1074	9.2%
Asthma	1922	7.3%	3065	22.1%	4554	10.9%	1745	5.9%	706	2.0%	139	1.2%
Urinary tract infections	848	3.2%	666	4.8%	4051	9.7%	1528	5.2%	2824	8.1%	1503	12.9%
Convulsions	3428	12.9%	1178	8.5%	2607	6.3%	1627	5.5%	1262	3.6%	426	3.6%
Chronic obstructive pulmonary disease	10	0.0%	0	0.0%	186	0.4%	2231	7.5%	5889	16.8%	1013	8.7%
Atrial fibrillation	0	0.0%	4	0.0%	363	0.9%	1525	5.1%	3225	9.2%	1027	8.8%
Epilepsy	408	1.5%	702	5.1%	2575	6.2%	1459	4.9%	761	2.2%	199	1.7%
Dental conditions	162	0.6%	368	2.7%	2863	6.9%	1094	3.7%	312	0.9%	44	0.4%
Congestive heart failure	10	0.0%	2	0.0%	76	0.2%	470	1.6%	2292	6.5%	1541	13.2%
Diabetes complications	48	0.2%	264	1.9%	1630	3.9%	988	3.3%	923	2.6%	230	2.0%
Flu and pneumonia	104	0.4%	49	0.4%	488	1.2%	678	2.3%	1397	4.0%	809	6.9%
Perforated ulcer	514	1.9%	53	0.4%	430	1.0%	322	1.1%	242	0.7%	62	0.5%
Hypertension	4	0.0%	11	0.1%	189	0.5%	438	1.5%	479	1.4%	141	1.2%
Dementia	0	0.0%	0	0.0%	1	0.0%	6	0.0%	140	0.4%	103	0.9%
Other vaccine preventable conditions	32	0.1%	20	0.1%	14	0.0%	8	0.0%	2	0.0%	1	0.0%
Anaemia	3	0.0%	2	0.0%	6	0.0%	5	0.0%	11	0.0%	8	0.1%

^a^ACSC: Ambulatory care sensitive condition.

As noted in Table 3, 55.0% of ACSC visits did not result in admission to hospital. Whilst the majority of ACSC visits were for acute rather than chronic conditions, acute visits were much more likely to conclude without admission (70.3% versus 33.4%). Visits for acute conditions were less likely to result in admission compared to chronic conditions across all age groups (Online Supplement 6 Table S6). For example, in patients aged 16 to 44, 77.8% of acute ACSC visits concluded without admission compared to 53.1% for chronic conditions. Whilst in patients aged 85+, 31.9% of acute visits concluded without admission compared to 17.5% of chronic conditions.

**Table 3.** Proportion of visits for ambulatory care sensitive conditions not admitted to hospital, by condition.

	Not admitted	% not admitted
Non-ACSC^a^	968,303	76.1
ACSC	87,089	55.0
Chronic ACSC	20,250	33.4
Acute ACSC	66,083	70.3
Vaccine preventable conditions	756	21.0
By condition		
Dental conditions	4510	93.1
Ear, nose and throat conditions	21,076	85.4
Perforated ulcer	1307	80.5
Other vaccine preventable conditions	60	77.9
Cellulitis	16,530	66.6
Dehydration and gastroenteritis	10,556	65.7
Urinary tract infection	7163	62.7
Hypertension	734	58.2
Epilepsy	3438	56.3
Asthma	6787	55.9
Anaemia	17	48.6
Convulsions	4941	46.9
Dementia	99	39.6
Atrial fibrillation	2395	39.0
Diabetes complications	1147	28.1
Chronic obstructive pulmonary disease	2298	24.6
Flu and pneumonia	696	19.7
Angina	2681	15.8
Congestive heart failure	654	14.9

^a^ACSC: Ambulatory care sensitive condition.

Returning to Table 3, the ACSCs for which visits most often concluded without hospital admission were dental conditions (93.1%), ear nose and throat infections (85.4%), perforated ulcer (80.5%), other vaccine preventable conditions (77.9%) and cellulitis (66.6%). Conversely, the ACSCs for which visits most often resulted in admission (the conditions that appear at the bottom of Table 3) were congestive heart failure (85.1% or 100%–14.9%), angina (84.2%), flu and pneumonia (80.3%), chronic obstructive pulmonary disease (75.4%) and diabetes (71.9%).

As noted in Table 4, there was a small difference by gender in the proportion of ACSC visits that concluded without admission (54.5% for men and 55.5% for women). ACSC visits were much more likely to conclude without hospital admission for younger patients (73.4% for patients aged 0–4, 71.8% for patients aged 5–15 years, and 70.8% for patients aged 16–44).

**Table 4.** Proportion of attendances for an ambulatory care sensitive condition not admitted to hospital, by patient characteristics.

	Not admitted	% not admitted
All ACSC^a^ attendances	87,089	55.0
Gender		
Male	43,724	54.5
Female	43,365	55.5
Age group		
Aged 0 to 4	19,445	73.4
Aged 5 to 15	9969	71.8
Aged 16 to 44	29,428	70.8
Aged 45 to 64	14,695	49.6
Aged 65 to 84	10,937	31.2
Aged 85+	2615	22.4
Major ethnic group		
White	62,229	52.8
Mixed	2657	69.2
Asian	9681	57.9
Black	3907	63.5
Other	2598	72.7
Unknown	6017	59.3
Index of multiple deprivation quintile		
Most deprived	34,242	56.7
2	15,586	55.0
3	12,938	54.3
4	11,550	52.0
Least deprived	11,781	53.4

^a^ACSC: Ambulatory care sensitive condition.

The highest proportion of ACSC visits that did not result in hospital admission were seen amongst patients of Other and Mixed ethnicity (72.7% and 69.2%, respectively). ACSC visits by Black patients concluded without admission in 63.5% of instances, and 57.9% of instances for Asian patients. White patients had the lowest proportion of ACSC visits not resulting in admission, in 52.8% of instances.

ACSC visits concluded without admission more often for patients living in the most deprived areas compared to patients living in the least deprived areas (56.7% versus 53.4%).

## Discussion

Emergency admissions for ACSCs are widely used as performance indicators, to determine where quality improvements are necessary and where best to direct resources. However, focussing only on admissions fails to account for a significant element of potentially preventable emergency care use. The majority of patients seeking care in an emergency do so by first attending the ED. When we examined patients attending the ED for an ACSC, we found that 55% of these visits did not conclude with hospital admission. Focussing only on ACSC admissions, therefore, misses over half of the instances in which patients accessed emergency care for a potentially preventable reason.

We found that 11.1% of all ED visits at the six hospital Trusts we analysed were preventable. In 2016/17 there were 15.5 million visits to type 1 EDs in England.^13^ If this 11.1% figure was applied to all hospital Trusts in England, this would suggest that 1.7 million potentially preventable ED visits occur annually, or approximately 31 potentially preventable visits per 1,000 population annually.

Acute conditions made up the majority of ACSC visits (60%), yet they had a much lower rate of admission to hospital than visits for chronic ACSCs. Widening the scope of current performance measures to examine ED visits, as well as admissions, would therefore reveal that a greater number of potentially preventable episodes of emergency care are the result of acute rather than chronic conditions.

The most frequent ACSCs among ED visits were cellulitis; ear, nose and throat infections; angina; dehydration and gastroenteritis; and asthma. However, the propensity to be admitted following a visit for an ACSC varies substantially between conditions, meaning that examining only emergency admissions for ACSCs provides a skewed picture of the types of conditions resulting in potentially preventable emergency care.

For example, ear, nose and throat infections were the second most frequent reason for an ACSC visit. However, 85% of these visits concluded without admission. Similarly, although dental conditions are a relatively rare presentation among ACSCs (3.1% of all presentations. see Table 1), visits for dental conditions conclude without admission to hospital in 93% of cases. Our results suggest that the focus only on admitted patients would underestimate the relative importance of such conditions in emergency care, and such conditions may warrant investigation and potential investment to improve the management of them in the community.

The propensity to be admitted following a visit for an ACSC was also found to vary substantially between patient groups. As expected, admission rates following ACSC visits were found to be higher in older patients. However, we also found the likelihood of admission following a visit for an ACSC to be highest for White patients, and lower for Black and Asian patients. The lowest proportion of ACSC visits resulting in admission to hospital was seen amongst patients of Other or Mixed ethnicity. Admission rates following visits for an ACSC were also lower amongst patients from the most deprived areas. Focussing only on admissions therefore disproportionately misses preventable episodes of emergency care experienced by young people, ethnic minority groups and more deprived populations.

### Comparison with previous literature

Whilst there is a large literature examining preventable health care utilization in terms of emergency admissions for ACSCs, few studies have extended the concept of ACSCs to ED visits. The findings and conclusions from international studies are highly dependent on the health care system. Those that have examined ACSCs in the context of the ED have done so in the US, where EDs often serve the role of a safety net providers and are often the main source of primary care for uninsured individuals or those that do not have a regular doctor,^20^ because services are provided regardless of insurance status or ability to pay.^21^ This is very different to their role in health care systems with universal coverage such as the UK. Therefore, in the US, ACSCs ED visits are used as an indicator of suboptimal access to primary care. However, in countries with universal health care such as the UK, where access to primary care is not determined by one’s ability to pay, the interest in these conditions is as measures of the quality of care delivered.^2^

Studies from the US suggest that ACSCs make up a substantial proportion of ED visits. However, prevalence estimates varied substantially by study setting. Johnson et al. found that 8.4% of ED visits by US adults were for ACSCs using survey data,^10^ Chukmaitov et al. estimated that 17.6% of ED visits in all Florida hospitals were for ACSCs,^11^ and Brownell et al.^12^ found that 28% of ED visits by elderly nursing home patients were for ACSCs.

An important reason for the difference in prevalence across studies is the use of different sets of conditions classified as ambulatory care sensitive. Any prevalence estimates will depend on the definition of ACSCs utilized, and there is no set of ACSC which is universally applied. Frick et al.^22^ examined the prevalence of ACSCs taken from various definitions amongst patients admitted to hospital from the ED, to determine the appropriateness of the conditions included in the definitions for use when analysing the ED population. They suggest the need for an ED optimised set of ACSCs, as some conditions seem particularly relevant for the population of patients attending the ED but are not included in most sets of ACSCs. In particular, they found that convulsions, urinary tract infections and atrial fibrillation are of particular relevance for the ED population but are excluded from most lists. These conditions are included in our definitions. However, Frick et al. analysed conditions amongst patients admitted from the ED, rather than all ED visits.

In England, a recent report found that ACSCs make up 23% of emergency admissions.^23^ This study used the same set of indicator measures to construct their list of ACSCs as in our analysis. The report showed that influenza and pneumonia, urinary tract infection, chronic obstructive pulmonary disease, dehydration and gastroenteritis and ear nose and throat conditions were the most frequent conditions amongst emergency admissions. The young and the elderly were shown to have the highest rates of ACSCs, and a strong correlation was found between deprivation and the rate of ACSC admissions. Another recent study found that several ethnic minority groups (Bangladeshi, Pakistani, Black African, White other or other background) had higher risks of ACSC admission compared to the White British majority group.^24^ Our results would suggest therefore that the disparities seen in the rate of ACSC admissions across these patient characteristics would be even more pronounced amongst ACSC visits.

### Limitations

This study adds to the recent growing literature highlighting the limitations of ACSCs admissions as indicators of the performance of primary care. One of the most notable limitations of using ACSCs as such an indicator is the inability to assess the preventability of individual presentations for ACSCs.^25^ One study found marked disagreement between admissions classified as preventable according to practice teams and the NHS list of ACSCs.^26^ Such limitations will also likely apply to ACSC ED visits. However, in the absence of methods to assess individual preventability, rates of emergency visits or admissions for ACSCs can still provide valuable area level information on preventable demand for care following adequate risk adjustment.^27^ In addition to these limitations we find that ACSC admissions also omit other important sources of preventable demand for emergency care.

Our study also has limitations. First, the experience at the six Trusts we looked at may not be generalizable to the whole country. This is the first study to estimate the prevalence of ED visits for ACSCs in England, using 2 years of patient-level data. We were able to do this by identifying six NHS hospital Trusts which utilize the ICD-10 diagnoses classification system in their ED records, therefore allowing for identification of visits for ACSCs. Although it is uncommon for diagnoses to be recorded in this way, the ICD-10 diagnosis classification is acknowledged as one of the diagnosis schemes hospitals are permitted to use in their ED records and is primarily a result of the computer software in use at the Trusts. Nonetheless, visits at these Trusts may not be representative of visits at all English hospitals. We compared characteristics of visits at these six Trusts to the rest of England and, encouragingly, we found broadly similar patterns with a slightly higher proportion of visits from patients living in the most deprived areas. However, none of the six Trusts are located in the north of England, whereas a recent report found that rates of admissions for ACSCs were higher in the north than the south of the country, after accounting for age, gender and deprivation.^23^

Second, our analysis was limited to visits at type 1 major EDs, and excluded visits at type 2–4 departments, which either offer specialised services or are intended for more minor complaints. Type 1 EDs are the most resource intensive of the emergency care facilities. Preventable demands placed on these services may therefore be diverting resources away from the most severely ill patients unnecessarily. Furthermore, this study only assesses emergency care sought through EDs. The majority of patients seeking care in an emergency do so by first attending the ED^28^; however, some emergency admissions will be admitted directly.

Third, there are limitations with ICD diagnosis codes. These codes are not used for reimbursement purposes in EDs in England therefore there is no financial incentive for accurate diagnosis record keeping. On average, ED diagnosis codes were missing in 25.3% of visits across EDs in England over the 2-year period we examine. To mitigate this issue, we limited our analysis to Trusts with levels of missing data below 12.5% for diagnosis codes. We checked the validity of the ICD-10 codes in the visit records and found that 99.93% of the codes were valid codes.

Furthermore, up to 12 diagnosis fields can be completed on the visit record,^18^ but we found that 94.54% of visits at the six Trusts in our analysis had just one diagnosis field completed. For the 5% of visits that do have more than one diagnosis field completed, we used the first diagnosis field (‘diag_01’) to classify a visit as ACSC, assuming that the first diagnosis is the primary reason for the visit. We examined the sensitivity of our results to this assumption, and found it made a negligible difference.

Moreover, not all patients attending an ED will receive a clear diagnosis during their visit. Patients attend with symptoms that will be investigated and treated, but a full diagnosis may not be formed whilst the patient is in the ED. Diagnosis fields in these cases may therefore represent presenting symptoms or the chief complaint of the patient. This is in comparison to admitted care, where diagnosis is made on the conclusion of the stay, when the clinical team have had more time to examine the underlying cause of a patient’s illness. Approximately, 18% of visits have a diagnosis recorded as an R chapter ICD code, which refer to symptoms, signs and abnormal clinical or laboratory findings, rather than a conclusive diagnosis. Therefore, our figures are likely to underestimate the true size of potentially preventable visits. Future research is needed to assess the extent of the problem posed by the reliance on ED diagnostic coding, by cross-referencing ED diagnostic coding with admitted care diagnosis using linked ED and admission records.

## Conclusions

Better management of patients with ACSCs has been a significant focus for health systems for many years, and admissions for ACSCs is a metric by which many organizations are monitored and compared internationally. Our results suggest that admissions for ACSCs do not provide the full picture when aiming to improve outcomes for patients. Whilst the cost consequences for the NHS of an ED visit are substantially lower than for an admission (average costs £148 vs £1590 in 2016/17),^29^ ED visits are experienced by a greater number of patients than hospital admission. Many ED visits do not result in admission to hospital, and these lower severity visits may be more sensitive to the quality and availability of services in the community. If we are concerned with preventing avoidable hospital use, then it is crucial to consider the potential to prevent ED visits in addition to admissions. Following on from this identification and descriptive analysis of ACSC ED visits, future research should focus on assessing the mechanisms that drive potentially preventable demand for emergency care, in order to inform policy.

To facilitate the development of a national performance indicator for ACSC visits, the recording of diagnoses in EDs would need to be standardised across all hospitals in the country, using more detailed diagnoses such as the ICD-10 classification system. A new emergency care data set has been introduced in England from October 2017,^30^ that requires the recording of ‘SNOMED’ diagnoses classifications, which are an even more granular and detailed classification system than the ICD.^31^ The identification of ED visits for ACSCs will therefore be possible on a national scale in the future. The calculation of such a measure would provide policy makers with a fuller picture of preventable emergency care, who experiences it, and for what illnesses. Our results suggest that it would likely highlight not only additional potentially preventable utilization, but different patterns of potentially preventable care – in terms of clinical conditions and patient groups – than those shown by existing measures based only on admissions. This could, in turn, identify new priority areas where emergency care use could potentially be prevented.

## Supplemental Material

sj-pdf-1-hsr-10.1177_13558196211059128 - Supplemental material for Unseen patterns of preventable emergency care: Emergency department visits for ambulatory care sensitive conditions Supplemental material, sj-pdf-1-hsr-10.1177_13558196211059128 for Unseen patterns of preventable emergency care: Emergency department visits for ambulatory care sensitive conditions by Beth Parkinson, Rachel Meacock, Katherine Checkland and Matt Sutton in Journal of Health Services Research & Policy

sj-pdf-2-hsr-10.1177_13558196211059128 - Supplemental material for Unseen patterns of preventable emergency care: Emergency department visits for ambulatory care sensitive conditions Supplemental material, sj-pdf-2-hsr-10.1177_13558196211059128 for Unseen patterns of preventable emergency care: Emergency department visits for ambulatory care sensitive conditions by Beth Parkinson, Rachel Meacock, Katherine Checkland and Matt Sutton in Journal of Health Services Research & Policy

sj-pdf-3-hsr-10.1177_13558196211059128 - Supplemental material for Unseen patterns of preventable emergency care: Emergency department visits for ambulatory care sensitive conditions Supplemental material, sj-pdf-3-hsr-10.1177_13558196211059128 for Unseen patterns of preventable emergency care: Emergency department visits for ambulatory care sensitive conditions by Beth Parkinson, Rachel Meacock, Katherine Checkland and Matt Sutton in Journal of Health Services Research & Policy

sj-pdf-4-hsr-10.1177_13558196211059128 - Supplemental material for Unseen patterns of preventable emergency care: Emergency department visits for ambulatory care sensitive conditions Supplemental material, sj-pdf-4-hsr-10.1177_13558196211059128 for Unseen patterns of preventable emergency care: Emergency department visits for ambulatory care sensitive conditions by Beth Parkinson, Rachel Meacock, Katherine Checkland and Matt Sutton in Journal of Health Services Research & Policy

sj-pdf-5-hsr-10.1177_13558196211059128 - Supplemental material for Unseen patterns of preventable emergency care: Emergency department visits for ambulatory care sensitive conditions Supplemental material, sj-pdf-5-hsr-10.1177_13558196211059128 for Unseen patterns of preventable emergency care: Emergency department visits for ambulatory care sensitive conditions by Beth Parkinson, Rachel Meacock, Katherine Checkland and Matt Sutton in Journal of Health Services Research & Policy

sj-pdf-6-hsr-10.1177_13558196211059128 - Supplemental material for Unseen patterns of preventable emergency care: Emergency department visits for ambulatory care sensitive conditions Supplemental material, sj-pdf-6-hsr-10.1177_13558196211059128 for Unseen patterns of preventable emergency care: Emergency department visits for ambulatory care sensitive conditions by Beth Parkinson, Rachel Meacock, Katherine Checkland and Matt Sutton in Journal of Health Services Research & Policy
