# Supplementary material for: Unseen patterns of preventable emergency care: Emergency department visits for ambulatory care sensitive conditions
Source: J Health Serv Res Policy. 2022 Feb 6;27(3):232–41. doi: 10.1177/13558196211059128 (PMC9277334; doi:10.1177/13558196211059128)
Supplement: sj-pdf-1-hsr-10.1177_13558196211059128 - Supplemental material for Unseen patterns of preventable emergency care: Emergency department visits for ambulatory care sensitive conditions [file sj-pdf-1-hsr-10.1177_13558196211059128.pdf]

Journal of Health Services Research & Policy
Unseen patterns of preventable emergency care: Emergency department visits for ambulatory care sensitive conditions
Beth Parkinson, Rachel Meacock, Kath Checkland and Matt Sutton

Online Supplement 1

Table S1 Ambulatory care sensitive conditions included in our analyses

Disease grouping	ICD-10 Code	Name
		Vaccine preventable conditions
Influenza and pneumonia	J10	Influenza due to identified influenza virus
	J11	Influenza, virus not identified
	J13X	Pneumonia due to Streptococcus pneumoniae
	J14	Pneumonia due to Haemophilus influenzae
	J15.3	Pneumonia due to streptococcus, group B
	J15.4	Pneumonia due to other streptococci
	J15.7	Pneumonia due to Mycoplasma pneumoniae
	J15.9	Bacterial pneumonia, unspecified
	J16.8	Pneumonia due to other specified infectious organisms
	J18.1	Lobar pneumonia, unspecified
	J18.8	Other pneumonia, organism unspecified
Other vaccine preventable conditions	A36	Diphtheria
	A37	Whooping cough
	B05	Measles
	B06	Rubella [German measles]
	B16.1	Acute hepatitis B with delta-agent (coinfection) without hepatic coma
	B16.9	Acute hepatitis B without delta-agent and without hepatic coma
	B26	Mumps
	M01.4	Rubella arthritis
	B18.1	Chronic viral hepatitis B without delta-agent
	B18.0	Chronic viral hepatitis B with delta-agent
		Acute conditions
Dehydration and gastroenteritis	E86	Volume depletion
	K52	Other noninfective gastroenteritis and colitis
	A02.0	Salmonella enteritis
	A04	Other bacterial intestinal infections
	A05.9	Bacterial foodborne intoxication, unspecified
	A07.2	Cryptosporidiosis
	A08	Viral and other specified intestinal infections
	A09	Gastroenteritis and colitis of infectious and unspecified origin
Urinary tract infections and Pyelonephritis	N10	Acute tubulo-interstitial nephritis
	N11	Chronic tubulo-interstitial nephritis
	N12	Tubulo-interstitial nephritis not specified as acute or chronic
	N13.6	Pyonephrosis
	N15.9	Renal tubulo-interstitial disease, unspecified
	N39.0	Urinary tract infection, site not specified
	N30.0	Acute cystitis

	N30.8	Other cystitis
	N30.9	Cystitis, unspecified
Perforated/ bleeding ulcer	K25.0-K25.2, K25.4-K25.6	Gastric ulcer
	K26.0-K26.2, K26.4-K26.6	Duodenal ulcer
	K27.0-K27.2, K27.4-K27.6	Peptic ulcer, site unspecified
	K28.0-K28.2, K28.4-K28.6	Gastrojejunal ulcer
	K20	Oesophagitis
	K21	Gastro-oesophageal reflux disease
Cellulitis	L03	Cellulitis
	L04	Acute lymphadenitis
	L08.0	Pyoderma
	L08.8	Other specified local infections of skin and subcutaneous tissue
	L08.9	Local infection of skin and subcutaneous tissue, unspecified
	L88	Pyoderma gangrenosum
	L98.0	Pyogenic granuloma
	I89.1	Lymphangitis
	L01	Impetigo
	L02	Cutaneous abscess, furuncle and carbuncle
Ear, nose and throat infections	H66	Suppurative and unspecified otitis media
	H67	Otitis media in diseases classified elsewhere
	J02	Acute pharyngitis
	J03	Acute tonsillitis
	J06	Acute upper respiratory infections of multiple and unspecified sites
	J31.2	Chronic pharyngitis
	J04.0	Acute laryngitis
Dental conditions	A69.0	Necrotizing ulcerative stomatitis
	K02	Dental caries
	K03	Other diseases of hard tissues of teeth
	K04	Diseases of pulp and periapical tissues
	K05	Gingivitis and periodontal diseases
	K06	Other disorders of gingiva and edentulous alveolar ridge
	K08	Other disorders of teeth and supporting structures
	K09.8	Other cysts of oral region, not elsewhere classified
	K09.9	Cyst of oral region, unspecified
	K12	Stomatitis and related lesions
	K13	Other diseases of lip and oral mucosa
	Chronic conditions	
Convulsions	R56	Convulsions, not elsewhere classified
	O15	Eclampsia
	G25.3	Myoclonus
Diabetes complications	E10	Insulin-dependent diabetes mellitus
	E11	Non-insulin-dependent diabetes mellitus

	E12	Malnutrition-related diabetes mellitus
	E13	Other specified diabetes mellitus
	E14	Unspecified diabetes mellitus
Anaemia	D50.1	Sideropenic dysphagia
	D50.8	Other iron deficiency anaemias
	D50.9	Iron deficiency anaemia, unspecified
	D51	Vitamin B12 deficiency anaemia
	D52	Folate deficiency anaemia
Dementia	F00	Dementia in Alzheimer disease
	F01	Vascular dementia
	F02	Dementia in other diseases classified elsewhere
	F03	Unspecified dementia
Epilepsy	G40	Epilepsy
	G41	Status epilepticus
Congestive heart failure	I11.0	Hypertensive heart disease with (congestive) heart failure
	I13.0	Hypertensive heart and renal disease with (congestive) heart failure
	I25	Chronic ischaemic heart disease
	I50	Heart failure
	J81X	Pulmonary oedema
Chronic obstructive pulmonary disease	J20	Acute bronchitis
	J41	Simple and mucopurulent chronic bronchitis
	J42X	Unspecified chronic bronchitis
	J43	Emphysema
	J44	Other chronic obstructive pulmonary disease
	J47X	Bronchiectasis
Asthma	J45	Asthma
	J46X	Status asthmaticus
Angina	I20	Angina pectoris
	I24.0*	Coronary thrombosis not resulting in myocardial infarction
	I24.8*	Other forms of acute ischaemic heart disease
	I24.9*	Acute ischaemic heart disease, unspecified
Atrial fibrillation	I48	Atrial fibrillation and flutter
Hypertension	I10X	Essential (primary) hypertension
	I11.9	Hypertensive heart disease without (congestive) heart failure

* These codes belong under Indicator 3.1 (acute) and have been included under the Angina heading when looking at conditions separately, but included as an acute presentation when comparing between acute and chronic conditions.
