# Supplementary material for: Unseen patterns of preventable emergency care: Emergency department visits for ambulatory care sensitive conditions
Source: J Health Serv Res Policy. 2022 Feb 6;27(3):232–41. doi: 10.1177/13558196211059128 (PMC9277334; doi:10.1177/13558196211059128)
Supplement: sj-pdf-2-hsr-10.1177_13558196211059128 - Supplemental material for Unseen patterns of preventable emergency care: Emergency department visits for ambulatory care sensitive conditions [file sj-pdf-2-hsr-10.1177_13558196211059128.pdf]

Journal of Health Services Research & Policy
Unseen patterns of preventable emergency care: Emergency department visits for ambulatory care sensitive conditions
Beth Parkinson, Rachel Meacock, Kath Checkland and Matt Sutton

Online Supplement 2

S2 Detailed description of the data cleaning process

In cleaning the diagnosis data for the six hospital Trusts included in our analysis, we removed additional characters from codes which were flagged by Stata's 'icd check' command as invalid, where the first three digits represented a valid three digit ICD code but the fourth character was either an 'X' (n=155,519 records) or additional digits which likely represented something specific to the NHS (n=8,181 records). For example, W19 is a valid three digit ICD code for an 'unspecified fall', whilst W199 appeared in 3,939 visits where the additional digit represents the location of the fall, in this case the 9 represents 'unspecified location'. The 'icd10 check' command identified the use of two ICD codes that were no longer valid for use in the 2010 version of the ICD, but represented ICD-10 codes previously valid in the 2008 version. These two codes were used in 1,253 visit records. We included these as valid codes for the purpose of our analysis. However, as neither of these two codes correspond to an ACSC, the only way that this impacted our analysis was in the assessment of the levels of missing data in the diagnosis code fields 24,409 visits had a diagnosis code of "R69" 'Unknown and unspecified causes of morbidity'.

Overall, 99.93% of non-missing diagnosis fields at these six hospital Trusts contained valid ICD-10 codes. For visits with an invalid ICD-10 diagnosis code, we set this to missing (n=960), which resulted in a total number of visits with missing diagnosis field information as n=75,776 (5.03% of total visits). Table S3 shows the level of missing diagnosis and output of 'icd10 check' for each trust.
