# Supplementary material for: Unseen patterns of preventable emergency care: Emergency department visits for ambulatory care sensitive conditions
Source: J Health Serv Res Policy. 2022 Feb 6;27(3):232–41. doi: 10.1177/13558196211059128 (PMC9277334; doi:10.1177/13558196211059128)
Supplement: sj-pdf-3-hsr-10.1177_13558196211059128 - Supplemental material for Unseen patterns of preventable emergency care: Emergency department visits for ambulatory care sensitive conditions [file sj-pdf-3-hsr-10.1177_13558196211059128.pdf]

Journal of Health Services Research & Policy

Unseen patterns of preventable emergency care: Emergency department visits for ambulatory care sensitive conditions

Beth Parkinson, Rachel Meacock, Kath Checkland and Matt Sutton

Online Supplement 3

Table S3 Missing diagnoses and ICD10 validity check by hospital Trust

	All 6 hospital Trusts combined		Trust 1		Trust 2		Trust 3		Trust 4		Trust 5		Trust 6	
	N	% of total visits	N	% of total visits	N	% of total visits	N	% of total visits	N	% of total visits	N	% of total visits	N	% of total visits
Total visits	1,505,979		143,960		145,960		258,860		215,680		528,660		212,860	
Missing diagnosis	74,816	4.97%	1,930	1.34%	0	0.00%	0	0.00%	8,755	4.06%	38,845	7.35%	25,285	11.88%
Non-missing diagnosis	1,431,163	95.03%	142,025	98.66%	145,960	100.00%	258,860	100.00%	206,920	95.94%	48,9815	92.65%	187,575	88.12%
ICD Check first diagnosis (diag_01)	N	% of non-missing	N	% of non-missing	N	% of non-missing	N	% of non-missing	N	% of non-missing	N	% of non-missing	N	% of non-missing
Defined code	1,428,950	99.85%	141,805	99.85%	145,655	99.79%	257,535	99.49%	206,750	99.92%	489,630	99.96%	187,575	100.00%
Code too short	1	0.00%	0	0.00%	0	0.00%	*	*	0	0.00%	0	0.00%	0	0.00%
Invalid 1st char (not A-Z)	281	0.02%	215	0.15%	0	0.00%	0	0.00%	50	0.02%	15	0.00%	0	0.00%
Invalid 2nd char (not 0-9)	673	0.05%	0	0.00%	0	0.00%	670	0.26%	0	0.00%	0	0.00%	*	*
Code not defined	5	0.00%	*	*	0	0.00%	0	0.00%	0	0.00%	0	0.00%	0	0.00%
Total invalid	960	0.07%	220	0.15%	0	0.00%	670	0.26%	50	0.02%	15	0.00%	*	*
	N	% of total	N	% of total	N	% of total	N	% of total	N	% of total	N	% of total	N	% of total
Missing + invalid diagnoses	75,776	5.03%	2,155	1.50%	0	0.00%	670	0.26%	8,805	4.08%	38,860	7.35%	25,285	11.88%
Non-missing + valid visits	1,430,203		141,805		145,960		258,190		206,870		489,800		187,575	

Due to HES data disclosure the trust level data volumes were rounded to nearest 5. Volumes of 7 or under, and their associated percentages were supressed, represented by *.
