# Supplementary material for: Unseen patterns of preventable emergency care: Emergency department visits for ambulatory care sensitive conditions
Source: J Health Serv Res Policy. 2022 Feb 6;27(3):232–41. doi: 10.1177/13558196211059128 (PMC9277334; doi:10.1177/13558196211059128)
Supplement: sj-pdf-4-hsr-10.1177_13558196211059128 - Supplemental material for Unseen patterns of preventable emergency care: Emergency department visits for ambulatory care sensitive conditions [file sj-pdf-4-hsr-10.1177_13558196211059128.pdf]

Journal of Health Services Research & Policy
Unseen patterns of preventable emergency care: Emergency department visits for ambulatory care sensitive conditions
Beth Parkinson, Rachel Meacock, Kath Checkland and Matt Sutton

Online Supplement 4

Table S4 Visit characteristics at the six hospital Trusts included in our analysis and all other hospital Trusts in England

	Six ICD Trusts	All other trusts
Total visits	1505979	29246267
Gender		
Male	759660 (50.4%)	14561258 (49.8%)
Female	746319 (49.6%)	14685009 (50.2%)
Average age	39.7 (26.7)	40.2 (26.7)
Age group		
0 to 4	154879 (10.3%)	3058426 (10.5%)
5 to 15	173413 (11.5%)	3127488 (10.7%)
16 to 64	842503 (55.9%)	16390589 (56.0%)
65 to 74	121808 (8.1%)	2413298 (8.3%)
75 to 84	124037 (8.2%)	2465663 (8.4%)
85+	89339 (5.9%)	1790803 (6.1%)
Arrival mode		
Brought in by ambulance	448124 (29.8%)	8753237 (29.9%)
Other	1057851 (70.2%)	20255197 (69.3%)
Not known	4 (0.0%)	237833 (0.8%)
Disposal after discharge		
Admitted to hospital	391322 (26.0%)	7807191 (26.7%)
Discharged - follow-up treatment to be provided by GP	233091 (15.5%)	5863759 (20.0%)
Discharged - did not require any follow-up treatment	588211 (39.1%)	9810677 (33.5%)
Referred to A&E clinic	19159 (1.3%)	394454 (1.3%)
Referred to fracture clinic	73048 (4.9%)	1220045 (4.2%)
Referred to other outpatient clinic	61470 (4.1%)	1283857 (4.4%)
Transferred to other healthcare provider	58126 (3.9%)	549697 (1.9%)
Died in department	1733 (0.1%)	35860 (0.1%)
Referred to other healthcare professional	19833 (1.3%)	631777 (2.2%)
Left department before being treated	42628 (2.8%)	967611 (3.3%)
Left department having refused treatment	9730 (0.6%)	156560 (0.5%)
Other	7486 (0.5%)	452866 (1.5%)
Not known	142 (0.0%)	71913 (0.2%)
IMD Score	27.5 (18.3)	25.2 (16.4)
IMD Quintile		
Most Deprived	531583 (35.7%)	7870701 (27.4%)
2	257123 (17.3%)	6509822 (22.7%)
3	235251 (15.8%)	5431008 (18.9%)
4	221590 (14.9%)	4759177 (16.6%)
Least deprived	244746 (16.4%)	4137733 (14.4%)
