# Supplementary material for: Unseen patterns of preventable emergency care: Emergency department visits for ambulatory care sensitive conditions
Source: J Health Serv Res Policy. 2022 Feb 6;27(3):232–41. doi: 10.1177/13558196211059128 (PMC9277334; doi:10.1177/13558196211059128)
Supplement: sj-pdf-5-hsr-10.1177_13558196211059128 - Supplemental material for Unseen patterns of preventable emergency care: Emergency department visits for ambulatory care sensitive conditions [file sj-pdf-5-hsr-10.1177_13558196211059128.pdf]

Journal of Health Services Research & Policy
Unseen patterns of preventable emergency care: Emergency department visits for ambulatory care sensitive conditions
Beth Parkinson, Rachel Meacock, Kath Checkland and Matt Sutton

Online Supplement 5

Table S5 Sensitivity analyses. Emergency department visits for ambulatory care sensitive conditions

	Sensitivity check 1: Restricting to visits with no secondary diagnoses		Sensitivity check 2 identification of ACSC based on ACSC in any diagnosis field on visit record	
	All 6 trusts		All 6 trusts	
	N	% of all visits	N	% of all visits
Total visits	1352135		1430232	
Non-ACSC	1203026	88.97%	1266176	88.53%
ACSC	149109	11.03%	164056	11.47%
	N	% of ACSC visits	N	% of ACSC visits
Chronic ACSC	56664	38.00%	62651	38.19%
Acute ACSC	89163	59.80%	98064	59.77%
Flu or vaccine preventable condition	3282	2.20%	4047	2.47%
By condition	N	% of ACSC visits	N	% of ACSC visits
Cellulitis	23925	16.05%	25797	15.72%
Ear nose and throat conditions	23486	15.75%	25438	15.51%
Angina	16224	10.88%	17152	10.45%
Dehydration and gastroenteritis	15096	10.12%	16687	10.17%
Asthma	11439	7.67%	12405	7.56%
Urinary tract infections	10719	7.19%	12265	7.48%
Convulsions	9792	6.57%	10853	6.62%
COPD	8435	5.66%	9695	5.91%
Epilepsy	5795	3.89%	6412	3.91%
Atrial fibrillation	5467	3.67%	6308	3.85%
Dental conditions	4634	3.11%	4923	3.00%
Diabetes complications	3925	2.63%	4585	2.79%
Congestive heart failure	3894	2.61%	4205	2.56%
Flu and pneumonia	3206	2.15%	3812	2.32%
Perforated ulcer	1511	1.01%	1768	1.08%
Hypertension	1232	0.83%	1326	0.81%
Dementia	218	0.15%	276	0.17%
Other vaccine preventable conditions	76	0.05%	84	0.05%
Anaemia	35	0.02%	36	0.02%

ACSC: Ambulatory care sensitive condition.
