# Supplementary material for: Unseen patterns of preventable emergency care: Emergency department visits for ambulatory care sensitive conditions
Source: J Health Serv Res Policy. 2022 Feb 6;27(3):232–41. doi: 10.1177/13558196211059128 (PMC9277334; doi:10.1177/13558196211059128)
Supplement: sj-pdf-6-hsr-10.1177_13558196211059128 - Supplemental material for Unseen patterns of preventable emergency care: Emergency department visits for ambulatory care sensitive conditions [file sj-pdf-6-hsr-10.1177_13558196211059128.pdf]

Journal of Health Services Research & Policy
Unseen patterns of preventable emergency care: Emergency department visits for ambulatory care sensitive conditions
Beth Parkinson, Rachel Meacock, Kath Checkland and Matt Sutton

Online Supplement 6

Table S6 Proportion of visits for ambulatory care sensitive conditions not admitted to hospital by condition

	Age 0 to 4	Age 5 to 15	Age 16 to 44	Age 45 to 64	Age 65 to 84	Age 85+
	% not admitted	% not admitted	% not admitted	% not admitted	% not admitted	% not admitted
Total	79.05%	89.20%	83.51%	73.45%	52.14%	36.08%
Non-ACSC	80.31%	90.79%	84.61%	76.36%	55.76%	38.27%
ACSC	73.44%	71.81%	70.75%	49.62%	31.25%	22.36%
Chronic	36.34%	48.53%	53.09%	34.65%	23.65%	17.49%
Acute	77.40%	81.54%	77.84%	66.85%	47.08%	31.87%
Vaccine preventable conditions	33.82%	62.32%	45.42%	29.74%	12.65%	7.16%
Dental conditions	93.21%	90.49%	94.06%	93.05%	90.71%	72.73%
Ear nose and throat conditions	87.08%	90.34%	81.21%	84.54%	66.54%	47.87%
Perforated ulcer	82.10%	88.68%	88.60%	79.81%	68.60%	54.84%
Other vaccine preventable conditions	71.88%	100.00%	78.57%	62.50%	50.00%	0.00%
Cellulitis	79.17%	81.43%	74.27%	66.87%	50.33%	33.04%
Dehydration and gastroenteritis	77.12%	83.28%	81.24%	58.32%	31.89%	14.62%
Urinary tract infections	69.81%	81.98%	74.43%	64.73%	49.61%	41.25%
Hypertension	50.00%	36.36%	58.73%	61.64%	58.66%	46.81%
Epilepsy	34.56%	46.58%	68.19%	57.64%	40.87%	31.16%
Asthma	36.89%	51.71%	68.03%	59.20%	44.90%	32.37%
Anaemia	100.00%	100.00%	83.33%	60.00%	36.36%	0.00%
Convulsions	41.66%	45.25%	64.21%	44.93%	36.29%	27.46%
Dementia	0.00%	0.00%	0.00%	50.00%	40.71%	37.86%
Atrial fibrillation	0.00%	50.00%	53.99%	48.39%	37.98%	22.78%
Diabetes complications	16.67%	17.42%	25.77%	35.43%	29.69%	21.30%
COPD	50.00%	0.00%	39.78%	33.75%	21.55%	19.45%
Flu and pneumonia	22.12%	46.94%	44.47%	29.35%	12.60%	7.17%
Angina	0.00%	33.33%	18.11%	18.35%	14.42%	10.62%
Congestive heart failure	60.00%	0.00%	14.47%	19.36%	14.62%	13.69%

ACSC: Ambulatory care sensitive condition
